# Optimisation: defining and exploring a concept to enhance the impact of public health initiatives

**DOI:** 10.1186/s12961-019-0502-6

**Published:** 2019-12-30

**Authors:** Luke Wolfenden, Katarzyna Bolsewicz, Alice Grady, Sam McCrabb, Melanie Kingsland, John Wiggers, Adrian Bauman, Rebecca Wyse, Nicole Nathan, Rachel Sutherland, Rebecca Kate Hodder, Maria Fernandez, Cara Lewis, Natalie Taylor, Heather McKay, Jeremy Grimshaw, Alix Hall, Joanna Moullin, Bianca Albers, Samantha Batchelor, John Attia, Andrew Milat, Andrew Bailey, Chris Rissel, Penny Reeves, Joanie Sims-Gould, Robyn Mildon, Chris Doran, Sze Lin Yoong

**Affiliations:** 1 Hunter New England Local Health District, Wallsend, NSW Australia; 2 School of Medicine and Public Health, University of Newcastle, Callaghan, NSW Australia; 3 Priority Research Centre for Health Behaviour, University of Newcastle, Callaghan, Australia; 4 Hunter Medical Research Institute, Newcastle, NSW Australia; 5 Faculty of Medicine and Health, University of Sydney, Camperdown, NSW Australia; 6 Centre for Health Promotion and Prevention Research, School of Public Health, University of Texas Health Science Centre, Houston, TX United States of America; 7 Kaiser Permanent Washington Health Research Institute, Seattle, WA United States of America; 8 Cancer Council NSW, Woollomooloo, NSW Australia; 9 Centre for Hip Health and Mobility, Robert H N Ho Research Centre, University of British Columbia, Vancouver, BC Canada; 10 Ottawa Hospital Research Institute, Ottawa, ON Canada; 11 Faculty of Health Sciences, School of Pharmacy and Biomedical Sciences, Curtin University, Perth, WA Australia; 12 European Implementation Collaborative, Sydney, Australia; 13 Central Coast Local Health District, Gosford, NSW Australia; 14 NSW Ministry of Health, North Sydney, NSW Australia; 15 Mid North Coast Local Health District, Port Macquarie, NSW Australia; 16 NSW Office of Preventive Health, Liverpool, NSW Australia; 17 Centre for Evidence and Implementation, Carlton, VIC Australia; 18 Central Queensland University, North Rockhampton, QLD Australia

**Keywords:** Optimisation, implementation, public health, intervention, Delphi study, consensus process, qualitative, adaptation, impact, evidence-based practice

## Abstract

**Background:**

Repeated, data-driven optimisation processes have been applied in many fields to rapidly transform the performance of products, processes and interventions. While such processes may similarly be employed to enhance the impact of public health initiatives, optimisation has not been defined in the context of public health and there has been little exploration of its key concepts.

**Methods:**

We used a modified, three-round Delphi study with an international group of researchers, public health policy-makers and practitioners to (1) generate a consensus-based definition of optimisation in the context of public health and (2i) describe key considerations for optimisation in that context.

A pre-workshop literature review and elicitation of participant views regarding optimisation in public health (round 1) were followed by a daylong workshop and facilitated face-to-face group discussions to refine the definition and generate key considerations (round 2); finally, post-workshop discussions were undertaken to refine and finalise the findings (round 3). A thematic analysis was performed at each round. Study findings reflect an iterative consultation process with study participants.

**Results:**

Thirty of 33 invited individuals (91%) participated in the study. Participants reached consensus on the following definition of optimisation in public health: “*A deliberate, iterative and data-driven process to improve a health intervention and/or its implementation to meet stakeholder-defined public health impacts within resource constraints*”.

A range of optimisation considerations were explored. Optimisation was considered most suitable when existing public health initiatives are not sufficiently effective, meaningful improvements from an optimisation process are anticipated, quality data to assess impacts are routinely available, and there are stable and ongoing resources to support it. Participants believed optimisation could be applied to improve the impacts of an intervention, an implementation strategy or both, on outcomes valued by stakeholders or end users. While optimisation processes were thought to be facilitated by an understanding of the mechanisms of an intervention or implementation strategy, no agreement was reached regarding the best approach to inform decisions about modifications to improve impact.

**Conclusions:**

The study findings provide a strong basis for future research to explore the potential impact of optimisation in the field of public health.

## Contributions to the literature

- This is the first study to generate a consensus-based definition of optimisation in the context of public health and to examine key considerations for optimisation in that context from the perspectives of researchers, public health policy-makers and practitioners.
- The study identified a number of seminal issues related to the application of optimisation processes, including whether, when and how such processes should be undertaken. We recommend further research be undertaken to investigate these issues explicitly and in more depth.
- The study findings provide a strong basis for future research toward the development of practical guidance to aid public health policy-makers and practitioners in their efforts to optimise the impact of public health initiatives.

## Background

Public health interventions are designed to address a range of modifiable risk factors of non-communicable disease; however, they often yield modest improvements in population health [1–4]. Furthermore, the effectiveness of interventions is often reduced as interventions are evaluated in more naturalistic contexts. For example, a systematic review of obesity prevention programmes found that those interventions tested in more real world (‘pragmatic’ trials) contexts did not significantly reduce child body mass index (− 0.09 kg/m^2^; 95% CI, − 0.19 to 0.01) while those under taken under more controlled research environments (explanatory trials) did (− 0.21 kg/m^2^; 95% CI, − 0.35 to − 0.08) [5]. Similarly, a meta-analysis of childcare-based physical activity intervention reported significant effects for trials evaluated under research conditions (SMD 0.80; 95% CI, 0.12 to 1.48) but not more real world environments (SMD 0.10; 95% CI, − 0.13 to 0.33) [4].

A number of factors have been suggested to contribute to the disappointing impact of many non-communicable disease interventions, particularly those evaluated in more naturalistic environments, including differences in the characteristics of participants and the availability of expertise and resources between efficacy research and evaluations undertaken in community contexts [4, 6]. Suboptimal implementation of interventions, however, has been frequently identified as a fundamental contributor to their variable and sometimes limited effect [7–9]. Implementation strategies are methods or techniques used to enhance the adoption, implementation and sustainability of an intervention [10]. They may include strategies such as educational meetings, audit and feedback, local technical assistance, or building coalitions [11]. However, reviews of the effects of such strategies indicate that, to date, they typically result in only small improvements in the fidelity of intervention implementation [10, 12–15]. Such findings have been consistent across clinical and community settings for a variety of public health and clinical conditions [12–15]. It is perhaps unsurprising then, that interventions of modest effectiveness, delivered in real-world contexts using strategies with modest impact on implementation may fail to achieve intended improvements in public health.

A further complicating factor to the translation of public health research evidence into community health improvement is that many tested public health interventions, and strategies to implement them, may not be suitable for widespread application in usual service delivery contexts [6]. As a result, adaptations are often made to ensure interventions and implementation strategies are suitable for the characteristics of the local population and can be delivered within the existing skills, resources and infrastructure of provider organisations [16]. While the process of ‘adaptation’ has been variously defined in the literature, broadly, it is understood to involve modifications to the intervention or to the approaches for their implementation to improve ‘fit’ with local contexts and capacity [17]. Reviews of the impact of adaptations, however, suggest that they can have a beneficial or detrimental effect on the impact of health initiatives [18].

While the purpose of adaptation is to improve ‘fit’, processes of repeated, purposeful modification (or adaptation), routinely occur in other fields, such as engineering and information technology, for the purpose of ‘optimising’ the performance of products through the accumulation of incremental improvements. Similar concepts are implicit in continuous quality improvement approaches in medical care [19, 20]. Ongoing, purposeful adaptations to interventions or implementation strategies may similarly represent a promising approach to ‘optimise’ the potential impact of public health interventions in achieving public health objectives. Such an approach may be particularly beneficial when undertaken in the context where the intervention is to be implemented and by, or in partnership with, the agency responsible for its delivery (and other end-users). It is also consistent with recommendations that health services generate and use data for service improvement [21].

There are a number of recent examples of systematic and iterative approaches to optimising the effectiveness of public health interventions and their implementation. The multi-phase optimisation strategy is a process recommended for developing and evaluating e-Health interventions through identifying and refining active intervention components and their dose prior to undertaking a confirmatory randomised trial [22]; it has been applied to a variety of public health issues, including obesity, smoking cessation and HIV to maximise the effects of these interventions [22–24]. Similar to the focus of quality improvement and continuous quality improvement methods in medicine [19, 20], other processes of iterative, data-informed modifications in public health have targeted the enhancement of the impact of implementation strategies. For example, sequential randomised evaluations of three strategies to implement school nutrition policies improved the incremental cost effectiveness ratios for implementation of the policy in schools (versus usual care) from $4730 to $2627 and facilitated its subsequent implementation ‘at scale’ [25, 26]. While such examples exist, optimisation processes applied across phases of intervention development to large-scale delivery appear uncommon. In Canada, for example, government and private foundations have funded thousands of public health pilot projects that are rarely further developed, improved and integrated into public health services – an outcome described by a former health minister as a tragic ‘waste of time, talent and energy’ [27]. Further, while evidence may be used to inform selection of public health interventions, the effectiveness of approaches to their implementation or their effects on community health outcomes once adopted as a health service are rarely evaluated, precluding the opportunity for ongoing, evidence-based evolution of the programme [28].

While examples of approaches to iteratively enhance the impact of a public health initiative exist, and employ similar methods, they do not appear to be bound by a unifying, clearly defined concept. Work in the area has also tended to focus either on improving the impact of an intervention or its implementation strategy. Both, however, represent important determinants of public health impact. To progress optimisation in public health, a standardised terminology is needed to provide clarity of concepts and facilitate communication and shared understanding among those working in interdisciplinary fields. It can also help avoid definitional issues that often plague emerging disciplines [29]. Defining the concept and key parameters of optimisation as it applies to public health, therefore, will provide a basis for subsequent work to develop conceptual understanding, methodologies, techniques, measures and practical guidance to advance the science of practice of optimisation for public health improvement. In in the context of public health we aimed to (1) generate a consensus-based definition of optimisation and (2) describe key considerations for optimisation.

## Methods

This was a pragmatic qualitative study using elements of participatory research and content analysis [30]. The three-round modified Delphi consensus development process [31, 32] used in this study was adapted to utilise literature reviews as well as multiple qualitative methods to elicit participants’ opinions and to facilitate group discussions. Question guides and materials were pilot tested with a group of public health research and implementation specialists and revised accordingly. The modified Delphi approach was used to address the first aim, while the qualitative elicitation methods, including those undertaken to establish a definition of optimisation, were used to address the second aim. The study was performed between September and December 2018. The study received low-risk ethical approval from the University of Newcastle’s Human Research Ethics Committee, Protocol No H-2018-0306.

### The selection of experts

The study used purposeful sampling to recruit two samples of participants representing two broad areas of implementation and public health expertise – (1) research and (2) policy and practice [32, 33]. Research experts included international implementation scientists, behavioural scientists, public health researchers, health service research economists and statisticians. Individuals with leadership roles in international public health, or implementation organisations or professional associations were prioritised. Policy and practice experts included public health policy-makers and practitioners from Australian government organisations. These groups were selected to capture the perspectives of key stakeholders involved in the development and implementation of public health interventions.

Experts were selected based on existing networks of the research team and were sent a formal invitation, study information (including the research team’s reasons and interests in the research topic) and consent form via email. Participation was required to attend a workshop in person hosted at the University of Newcastle, New South Wales (NSW) Australia.

The sample size was guided by a similar modified Delphi study [32] and recommendations for qualitative group discussions [30, 33]. Such recommendations suggest a minimum of two focus groups (four to eight people in a group) of participants representing similar characteristics (research experts, policy and practice experts) [30, 33]. Based on such recommendations, we required a minimum sample size of 30 participants.

### Delphi rounds

**Fig. 1:**
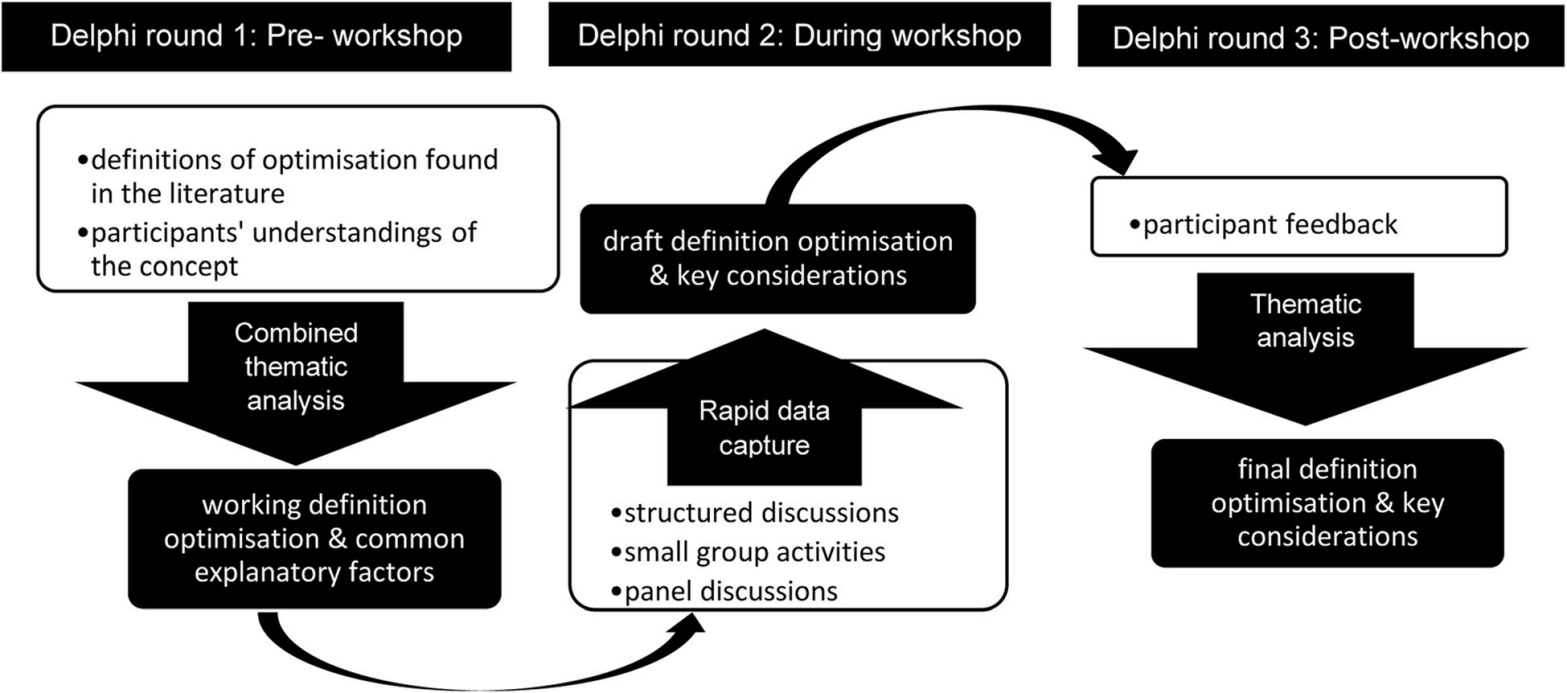
Modified Delphi consensus process used in the study

The first Delphi round was undertaken prior, the second was undertaken during, and the final was undertaken following the workshop. Activities undertaken at each round of the consensus process (including methods to capture and analyse data at each point) are described below and summarised in Fig. 1.

#### First round Delphi study – pre-workshop

Two weeks prior to attending the workshop, participants were emailed the following request “*In the context of public health and health promotion intervention development and implementation, please describe your understanding of the term ‘optimisation’*”*.* The expression of the request was developed based on that employed by Milat et al. [32] in their Delphi study used to define scalability in the context of public health. Participants were asked to provide a free text response via email.

Concurrently, the research team undertook a scoping literature review following the Johanna Briggs Institute method [34] to identify frameworks relevant to the concept of optimisation. A scoping review is a form of systematic review conducted when there is uncertainty in the literature to examine the key themes, concepts and definitions relating to an area of research [34]. The scoping review ensured that the study identified prior work in the literature relevant to optimisation for participants to make use of and consider in the development of a definition. To be eligible, manuscripts needed to include a definition of optimisation or quality improvement. We systematically searched MEDLINE, CINAHL, PsycINFO and ProQuest Nursing & Allied Health Source databases combining terms for framework, health context and improvement (Additional file 1). Key publications known a priori and references of key publications were also searched for relevant definitions.

Existing definitions of optimisation or quality improvement were extracted from identified articles. Literature-derived definitions of optimisation and participants’ responses to the emailed request were then examined using a combined qualitative thematic analysis [30]. Four public health researchers (KB, SM, AG and SLY) collaboratively identified key themes across each of the literature-derived definitions and participants’ responses. The researchers then drafted two separate conceptual maps that captured and organised the themes (Additional files 2 and 3). A third combined conceptual map was conceived to reflect the key overlapping themes across the individual conceptual maps (Additional file 4) and served as a basis for drafting a definition for optimisation in public health. Within the third conceptual map, colours represented the source of the theme, that is, yellow represented the themes derived from the literature review, purple from the participant responses, and green the key overlapping themes across both the literature and participant responses.

#### Second round Delphi study – during the workshop

The second round Delphi took place during the face-to-face workshop (6 hours with meal breaks). The findings from the combined pre-workshop analysis conducted by the research team were presented using printed conceptual maps and two 15-min presentations (on round one Delphi and examples of optimisation in practice) by a member of the research team (AG). The draft definition was displayed in editable hard copy on the wall, and participants were encouraged to suggest changes to wording or make other notes or comments they believed should be considered in refining the definition at any time during the workshop.

Initial verbal feedback was solicited from all participants and, subsequently, the research team (LW, SLY) facilitated a whole group discussion to explore aspects of the definition. Participants were then divided into small groups to elicit additional feedback regarding the proposed working definition (KB). These discussions, thoughts or feedback were noted by small groups on ‘flipcharts’, and then presented back to the group. This was followed by a formal facilitated 30 min-long panel discussion (LW) focussing on practical considerations of optimisation, such as how, when and on what outcomes interventions or implementation strategies should be optimised. The panel was comprised of two researchers and two public health policy-makers and practitioners experienced in optimising in public health.

During the workshop, data were captured via structured notes by two trained note-takers and notes drafted onto flipchart paper by participants. Photos of participants’ contributions were taken. Rapid preliminary thematic analysis of this data was undertaken [17, 18] by four members of the research team (LW, SLY, AG, KB). This involved the research team gathering at interim sessions and reading through notes (taken by note-takers and participants), incorporating participants’ feedback into the definition of optimisation, and highlighting key emerging themes related to optimisation. A revised definition of optimisation was drafted and presented to participants at the conclusion of the workshop. The group discussed issues around the scope of the definition, its intended interpretation and other key considerations.

Immediately after the workshop, a research team member with a doctoral-level qualification in qualitative research (KB) consolidated all notes and photos from the workshop and organised them in qualitative software package NVivo 12 (QSR, Victoria, Australia). The preliminary data analysis initiated during the workshop was followed by a formal five-step process of thematic analysis that included (1) reading, (2) coding, (3) displaying, (4) reducing and (5) interpreting the data [33]. More specifically, KB read and, in consultation with the research team, developed memos and a coding tree, and then coded the data into broad themes and sub-themes corresponding to various topics of discussion, noting preliminary relationships between them. While some themes were identified in advance, some others were derived from the data [30]. The preliminary structure together with consolidated notes and photos from the workshop was then prepared for the final round Delphi study.

#### Final round Delphi study

The final round Delphi sought consensus on the proposed final definition of optimisation based on synthesis of feedback received during previous rounds. Participants received in email a draft document summarising the above and were invited to either approve the existing text of the definition, or to provide final feedback. A few minor changes to text were suggested, and once these were incorporated, the final definition was agreed to by all via email.

Throughout all rounds, refining of themes, reducing data into essential concepts and relationships, and interpretation of findings were done iteratively in partnership with the research team and participants. Using a team approach, being sensitive to divergent views and opinions, and having a clear record of verbal and written contributions enhanced the rigour of qualitative analysis and interpretation [35].

## Results

### Response rates and respondent characteristics

Thirty of 33 invited individuals (91%) provided active consent and participated in the study. Participants included men (*n* = 11) and women (*n* = 19), policy and practice experts (*n* = 16) and research experts (*n* = 14). Workshop attendees held appointments across seven universities or research institutes internationally (Table 1). Non-participants lived outside Australia and were not able to attend the workshop due to competing demands.

**Table 1:** Institutions represented at the workshop

Academic institutions	The University of Texas The University of Newcastle The University of British Columbia The University of Sydney Central Queensland University Curtin University
Professional associations	Cochrane; The Campbell Collaborations Knowledge Translation and Implementation Group; The Society for Implementation Research Collaboration; The University of Texas Centre for Health Promotion and Prevention Research; The European Implementation Collaborative; World Health Organization Collaborating Centre on Nutrition, Physical Activity, and Obesity; The Centre for Evidence and Implementation; The Australian Prevention Partnership Centre; Hunter Medical Research Institute; Cancer Council NSW; Ottawa Hospital Research Institute; Centre for Evidence and Implementation
Individual local health districts	NSW Hunter New England; Central Coast and Mid-North Coast Local Health Districts
Individual ministries of health	The NSW Ministry of Health; The NSW Office of Preventive Health

### Aim 1: Defining optimisation in the context of public health

**Fig. 2:**
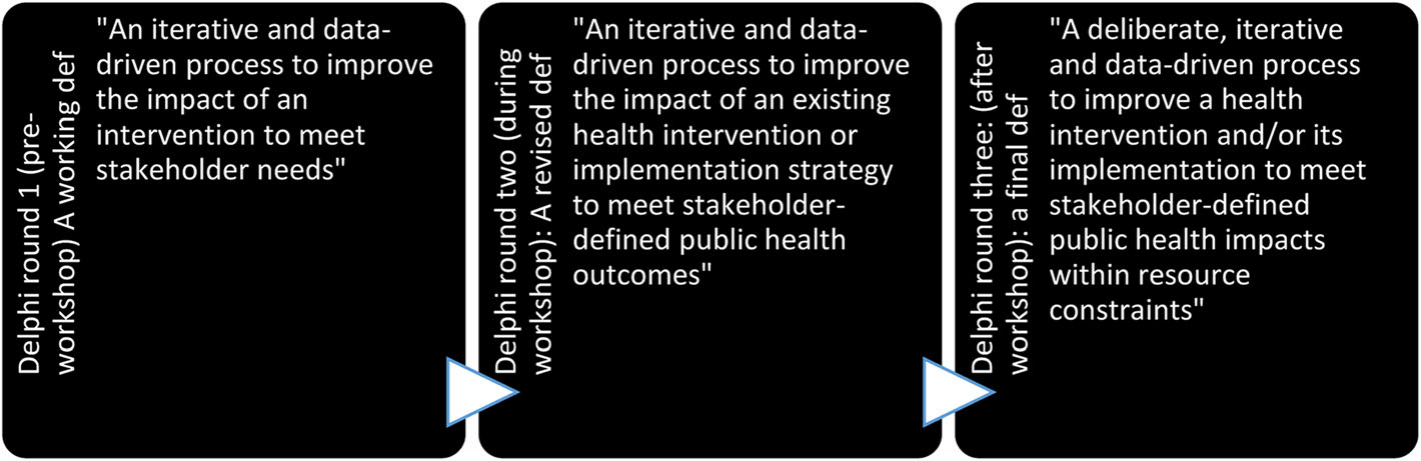
Stages of definition refinement

During subsequent Delphi rounds, the working definition of optimisation underwent several modifications (Fig. 2). Following the final round, the agreed upon definition of optimisation in the context of public health was*:* “*Optimisation is a deliberate, iterative and data-driven process to improve a health intervention and/or its implementation to meet stakeholder-defined public health impacts within resource constraints*”.

### Aim 2: Key considerations in optimisation for public health

Aligned with the second aim, over the course of the workshop, the participants discussed conceptual and practical considerations when optimising public health interventions. These were grouped into major themes and corresponding sub-themes, which are discussed in the following section, and summarised in Table 2.

**Table 2:** Key considerations when optimising public health interventions

Major theme	Sub-themes
Theme 1: Parameters for optimisation such as pre-conditions for optimisation and factors considered following a decision to optimise (when and on what outcome to optimise)	• Pre-conditions for optimisation: 1) Good quality outcome data and the resources to analyse/evaluate programme outcomes are available 2) Existing initiatives are not sufficiently effective and meaningful public health impacts are anticipated from optimisation 3) Organisational support and leadership for activities such as end-user engagement is available • Parameters considered following a decision to optimise (when and on what outcome to optimise): 1) Optimisation processes may occur across the public health translation continuum (intervention development through implementation at scale) 2) Optimisation should seek to improve impact on outcomes defined and valued by stakeholders (or end-users) 3) The impacts of optimisation are considered relative to the available resources
Theme 2. How to optimise	• The underlying initiative’s logic or causal model needs to be understood • Factorial designs or analogue methods may be used to understand the initiative’s mechanisms
Theme 3. Identifying when optimisation has been achieved	• Stakeholder views, potential for additional worthwhile impacts and balancing multiple outcomes need to be considered

#### Theme 1: Parameters for optimisation

Participants acknowledged there were various parameters of optimisation that needed to be taken into account either when considering whether to optimise or once a decision was made to proceed with optimisation. Parameters used when deciding whether to optimise include a range of pre-conditions that may need to be present for optimisation to be possible or potentially worthwhile. A different set of parameters may need to be used once a decision to optimise has taken place. These appear more focussed on what outcome and how the optimisation process should take place.

##### Pre-conditions for optimisation

1. Good quality outcome data and the resources to analyse/evaluate programme outcomes are available

The availability of good quality data for assessing the impact of the intervention and/or implementation strategy on the outcome for which it is being optimised is necessary for optimisation. Some participants commented that optimisation is particularly suited to interventions where there are routine data collected that can be inexpensively accessed and used to assess the impacts of interventions as they are iteratively modified. For example, existing medical records, linked data sets or data from public health chronic disease risk surveillance systems could be used for that purpose. However, participants noted the limitations of many of these data sources to provide sufficiently valid measures of improvement, and a considerable challenge that goes with identifying easily accessible, suitably robust measures for optimisation in public health.

Furthermore, given that optimisation processes may take considerable time (perhaps many years), the availability of ongoing stable resources was considered an important pre-condition for optimisation. Participants acknowledged that, while many public health services have ongoing recurrent funding for the delivery of health programmes and their evaluation, many others do not. In such circumstances, where only short-term discrete funds are available, optimisation for interventions or strategies to implement them may not be possible or appropriate.

2) Existing initiatives are not sufficiently effective and meaningful public health impacts are anticipated from optimisation

Participants discussed that, in order to warrant engaging in optimisation processes, there needs to be enough evidence to demonstrate that existing intervention or implementation approaches are not sufficiently effective. Additionally, meaningful improvements must be anticipated from an optimisation process to justify proceeding with optimisation.

“[to optimise] *you must have a problem worth solving*.” (Implementation scientist)

“*Consider minimum standard… avoid trying to optimise things that are not worth it.*” (Implementation scientist)

In other words, in order to embark on an optimisation process, the public health importance of the issue and the potential benefits of an optimised intervention/implementation strategy need to be weighed. Tobacco use, being highly prevalent in the community and responsible for considerable harm, is an example of a public health issue where improvements in impact could be expected over time through optimisation.

3) Organisational support and leadership for activities such as end-user engagement is available

Optimisation requires end-user engagement. Some participants suggested that engagement in optimisation processes may be a substantial investment that requires organisational support and commitment. Furthermore, on-going optimisation of an implementation strategy may be disruptive for the agency responsible for programme implementation. In such circumstances, organisation leadership and support and a readiness and willingness for ongoing change within the relevant organisations appear fundamental.

##### Parameters considered following a decision to optimise (when and on what outcome to optimise)

In addition to highlighting various pre-conditions for optimisation, the group discussed the types of parameters that might be considered once a decision was made to proceed with optimisation.

1. Optimisation processes may occur across the public health translation continuum (intervention development through implementation at scale)

The group debated whether optimisation processes could occur at any stage of the public health translation continuum – during intervention development, implementation strategy development, active dissemination and implementation at scale – and the types of data that are needed for different stages.

“*Need to consider that maybe optimisation is an ongoing process, not just at specific time points.*” (Practitioner)

“*May be optimisation isn’t at the end or beginning but goes throughout?*” (Epidemiologist)

Nonetheless, there were various and divergent views about the relative value of earlier stage optimisation processes applied to the development of interventions, and later stage optimisation processes that may be more focused on optimising its implementation in real-world contexts to maximise its impact. Some participants questioned the difference between optimisation during the early stages of intervention development and conventional formative research.

“*Is there anything you would do differently for a health promotion programme (i.e. good formative evaluation) to optimise pre-intervention, besides good planning?*” (Public health/behavioural scientist)

“*The distinction between good design… why make it cover things that are already well covered?*” (Practitioner)

Others proposed that a key difference was a specific focus of optimisation on improving impact, the purpose of optimisation being aligned to stakeholders’ goals rather than academic or programme developer goals, and the focus on continuous or ongoing improvement.

“*Optimisation goes beyond good planning…because in the process of optimising we are trying to get higher effectiveness…greater efficiency*.” (Practitioner)

The discussion concluded with a statement that, while optimisation processes may occur at any stage of the development and implementation of an intervention, the impact of intervention is more likely to be maximised if optimisation occurs throughout the public health translation continuum.

2) Optimisation should seek to improve impact on outcomes defined and valued by stakeholders (or end-users)

Participants considered the primary objective of optimisation to be an improvement in outcomes defined and valued by stakeholders, including cost, efficiency, alignment with existing programmes, and/or reach [36]. Typically, stakeholders are agencies or organisations responsible for financing the implementation of an intervention, or those responsible for intervention delivery. Depending on the stakeholders involved and the impact they seek to optimise, the processes and outcomes of optimisation may vary. On this basis, the group agreed that it was important first to define who the stakeholders for optimisation were.

When considering stakeholders, participants highlighted a need to differentiate between policy-makers (who could include government or non-government decision-makers) and end-users/consumers (who could include community members, patients and organisations such as health services schools or community organisations).

“*Start with stakeholders… optimise needs for stakeholders… policy-makers versus consumers. Have we met stakeholders’ needs? What are their needs*?” (Public health/behavioural scientist)

Making it clear who stakeholders are and how they differ may then help in establishing how to engage with each group in the public health implementation continuum.

Participants acknowledged that various and often divergent stakeholder perspectives and priorities need to be considered. Outcomes of optimisation could include measures of intervention effects on quality of care, individual health behaviours, conditions or quality of life, and population or health system level outcomes including measures of inequality, implementation, health service use or costs.

There was a general agreement that, in the context of public health, while there may be system-level outcome expectancies of policy-makers and/or researchers and organisational-level outcome expectancies of agency leaders and service providers, the micro-level outcome expectancies and priorities of end-users (such as reach, equity, appropriateness and quality of life) should always be considered.

“*Optimisation* [in public health] *comes with a positive intent*” (Implementation scientist)

*“For example, … optimising care for hip fracture patients. System optimised for cost, reduction in infection* [system level] *but not for quality of life for individual. Need to consider quality of life. Frameworks currently don’t cover that*” (Public health/behavioural scientist)

It seems that, in optimising public health interventions, it is paramount to meet end-user priorities and not to lose the focus from the ‘positive intent’ of optimisation.

“*Prime motivation is health of the population. This needs to be at the forefront of planning*” (Health service manager)

3) The impacts of optimisation are considered relative to the available resources

The impacts of optimisation occur in the context of and considered relative to the availability of finite resources.

“*Resource use is the key ingredient to undertaking activity*” (Methodologist)

Participants acknowledged that resource requirements are an important consideration across the whole process of optimisation in public health and that such requirements may differ at different phases of intervention development and implementation.

“*(…) while at the research phase we may be considering high risk/high yield; at the service delivery phase you may start with more resources than possible and then scale it for real world; and at the population level we need to consider effectiveness and population vulnerability*” (Implementation scientist)

The various parameters discussed above influence the process and outcomes of optimisation in public health. Participants also discussed some considerations around the potential methods to identify which elements of the programme to optimise.

#### Theme 2: How to optimise

A discussion around how to undertake optimisation reflected participants’ different perspectives, and no agreement was reached regarding a ‘best approach’ to identify how an intervention or implementation strategy should be modified in order for optimisation to occur.

##### The underlying initiative’s logic or causal model needs to be understood

Participants agreed that, in order to optimise, the underlying core components and mechanisms of a programme need to be understood. It was noted, however, that many programmes do not have a logic model and that there are scientific and practical considerations that limit opportunities and capacity to test hypothesised mechanisms.

“*Despite the fact that programme logic is so important for a proper programme evaluation, most programmes have no programme logic… we have no idea about what programme components address what aspects of the programme*.” (Practitioner)

##### Factorial designs or analogue methods may be used to understand the initiative’s mechanisms

Some suggested to optimise elements of the intervention or implementation strategy using quantitative mechanistic methods (e.g. mediation analyses) and research designs, including randomised and factorial trials as well as other more pragmatic approaches such as analogue methods (i.e. vignettes). Participants noted that analogue methods would help avoid the need to test the whole intervention in a large randomised control trial, as they would help determine – on a small scale – the factors that are influencing impacts.

“*We could use norms-based interventions to improve delivery… optimisation in an analogue or vignette space to test intervention with intended end-users, for example, testing how to more effectively deliver/communicate messages to end-users*…” (Implementation scientist)

#### Theme 3: Identifying when optimisation has been achieved

The final group of considerations around optimisation was related to how to know when an intervention and/or implementation strategy has been optimised.

##### Stakeholder views, potential for additional worthwhile impacts and balancing multiple outcomes need to be considered

Participants discussed the relationship between the parameters mentioned above (the priorities or outcome expectancies defined by stakeholders involved and resource constraints) and the decision regarding the point at which the programme (intervention or its implementation) is deemed ‘optimised’. No agreement was reached regarding a precise decision point or criteria. Specifically, some participants stressed that the values and perspectives of stakeholders need to be considered when deciding whether a programme has been optimised. Others suggested that optimisation has been reached when stakeholders consider that further investment in optimisation may not yield worthwhile improvements in impact. Furthermore, some participants related to the point of optimisation more conceptually, as a ‘balance point’. That ‘balance point’ would be between the acceptability to stakeholders and dimensions of a programme such as cost-effectiveness, budget impact, reach and effectiveness — understood within the specific context’s constraints.

## Discussion

To our knowledge, for the first time, this study provides a consensus-based definition of optimisation in the context of public health. It did so by employing the expertise of a group of international researchers, public health policy-makers and practitioners representing leading organisations across a range of disciplines. The key elements of the final consensus-based definition of optimisation were it being a process that was data driven, iterative, targeting an impact that is stakeholder defined and conducted in the context of finite health resources. Such elements align well with the evidence-based medicine paradigm [37], suggesting that the process is consistent with the underlying values of the field and may represent a promising approach of improving the health and welling of the community. Importantly, the study also explored seminal issues related to the application of optimisation in public health, including whether, when and how such processes should be undertaken. In doing so, the study provides greater conceptual clarity and a broad base for further work in the field.

A number of aspects of the definition are similar to optimisation processes in other fields [38–41], in particular its iterative and data-driven nature. There are also parallels to related concepts such as quality improvement cycles and other improvement frameworks in healthcare [19, 20]. The practice of optimisation is also not new in public health. There are a number of examples in public health of processes that would be consistent with the definition of optimisation proposed in this study [22–26]; however, these have typically focussed on approaches to improve the effectiveness of interventions during the intervention development stage or approaches to improve the effectiveness of strategies to improve programme implementation. A definition encompassing a range of stages of the translation continuum from intervention development to large-scale implementation appears unique in the context of public health and may provide a unifying concept for current work in the area. The explicit role of stakeholders in defining optimisation impacts may also be a distinguishing feature of optimisation relative to other related concepts in the field [42].

Participants identified a number of challenges to optimisation in public health that need to be considered prior to embarking on optimising, including the availability of good quality data to optimise implementation strategies for existing evidence-based interventions and the stability of funding to enable optimisation to occur over long periods. As such, there appears most opportunity for optimisation when the outcomes assessed can make use of routinely collected data sets such as administrative records, clinical records, public health surveillance systems or information technology. For optimisation processes to flourish in public health, novel methods of data capture or identifying sources of routinely collected robust outcome data will likely be required. Optimisation is also difficult if the underlying core components and mechanisms of a programme are not known or made explicit. In a field such as implementation science, there remains very little empirical evidence to support an understanding of implementation processes and impacts [43, 44]. Advances in mechanistic evaluation of implementation strategies will improve the viability of optimisation processes applied to implementation strategies in public health.

Nonetheless, the findings of this study suggest that the broad application of optimisation processes in public health is likely to represent a considerable challenge. As well as the practical considerations identified by participants, including access to routinely collected data, the public health workforce may require significant capacity-building or processes to engage those with expertise in health economics, research trial methods, mechanistic programme evaluation, adaptive interventions and research designs. Examples of where optimisation has been applied to improve the impacts of public health initiatives have typically been in the context where such expertise is available and has been applied [26, 45]. As such, strategies to support partnerships between researchers and public health policy-makers and practitioners, including embedding of researchers in public health service agencies, may represent one means of enhancing expertise, capacity and infrastructure to facilitate optimisation. Furthermore, public health decision-making is influenced by a range of social, political and organisational factors, of which research evidence is one [46]. Optimisation, particularly of public health policy, may be difficult to achieve in the context of these other considerations, which may favour policy stability (rather than change), the introduction of ‘new’ programmes (rather than optimisation of existing programmes) or investments in public health programmes that are short term. While the challenges are considerable, optimisation processes offer enormous potential to efficiently and expediently improve the impact of public health initiatives.

There are also some methodological aspects of the study that warrant consideration. The modified three-round Delphi approach with a highly interactive face-to-face component [47] was found appropriate to address the study aims. The day-long workshop was found particularly useful in engaging multiple stakeholders. The workshop also enabled multiple qualitative techniques to be applied, which was instrumental in eliciting participants’ opinions and gathering rich qualitative data that reflected both the individual contributions and the opinions that were formulated via group processes. We suggest that a traditional Delphi survey method would not have produced the highly nuanced data we were able to collect, or the type of evidence to question and expand on existing definitions of optimisation.

Participants were purposefully sampled to provide diverse expertise and broad representation of relevant public health professional associations, using existing networks of the research team to identify individuals that were well positioned to provide input into the research. It is possible that some participants may not have felt able to express their views freely if they had an existing professional association with a member of the research team. However, the extent to which this may have occurred and any bias it may have introduced are unclear. Nonetheless, participants arrived at a consensus definition of optimisation. A further limitation of the study was that it explored a number of key issues and concepts but, in many instances, this occurred at a high level. Furthermore, several discussions, such as methodological considerations in defining the outcome of optimisation and the levels at which optimisation in public health may occur (micro, meso and macro), were initiated but not well developed. These emerging topics were relevant to study participants and may warrant further investigation.

## Conclusions

The study highlighted the strength of engagement on the topic among public health experts in implementation science and practice. A consensus-based definition of optimisation in the context of public health was achieved, and various conceptual and practical considerations that accompany designing and executing optimisation in practice were mapped. Participants, representing global expertise in the field, expressed a strong interest in further exploring optimisation considerations that were discussed as part of the study as well as those that were recognised as research gaps. This highlights the importance and timeliness of the topic and its further exploration. Previous cases of optimisation in public health, for example, have demonstrated that, through repeated data, driven improvement, the cost of delivering effective public health programmes can be achieved at approximately half that of usual practice, effectively doubling its population level impact [26, 45]. The findings of this study suggest that, while representing a challenge, the concept of optimisation is relevant to public health and could be a particularly useful means of improving the impact of public health initiatives. The definition and early concepts regarding optimisation of public health provide a strong basis for future research to explore the potential impact of this promising approach in the field.

## Supplementary information

**Additional file 1.** Search terms.

**Additional file 2.** Conceptual map of 'Optimisation' derived from the literature.

**Additional file 3.** Conceptual map of 'Optimisation' derived from participant responses.

**Additional file 4.** A combined conceptual map highlighting the overlap between two individual maps.

**Additional file 5.** COREQ Checklist for qualitatitive studies.

## Data Availability

The datasets used and/or analysed during the current study are available from the corresponding author on reasonable request.

## Abbreviations

CI: confidence interval
SMD: standardised mean difference
