# Supplementary material for: Optimisation: defining and exploring a concept to enhance the impact of public health initiatives
Source: Health Res Policy Syst. 2019 Dec 30;17:108. doi: 10.1186/s12961-019-0502-6 (PMC6937822; doi:10.1186/s12961-019-0502-6)
Supplement: Supplementary file 1 — Additional file 1. Search terms. [file 12961_2019_502_MOESM1_ESM.docx]

Search terms and key publications whose references were searched, used in the scoping literature review to identify frameworks relevant to the concept of optimisation.

Search terms for frameworks were developed based on terminology used by Levati et al.^6^ (optimisation), Kaplan et al.^20^ (QI), Gardner et al.^15^ (CQI), Kaplan et al.^20^ and Gardner et al.^15^ (health context), Mouillin et al.^16^(frameworks) and Mouillin et al. and Gardner et al.^15, 16, 19^ (pre-identified frameworks). Research by Hempel et al. (2011)^21^ showed that the yield from using more complex search terms for QI intervention may be unmanageable and non-essential.

**Table 2.** Search terms (first round)

| **Improvement terms (Optimisation, QI, and CQI)** | **1** | (“quality improvement*” or “improve* quality” or “quality management*” or “improve* patient care” or “process improvement”).ti,ab. |
| --- | --- | --- |
|  | **2** | “Continuous Quality Improvement”.ti,ab. |
|  | **3** | “Total quality management”.ti,ab. |
|  | **4** | (“optimisation” or “optimization” or “optimi*”).ti,ab. |
|  | **5** | Or/1-4 |
| **Context** | **6** | (community or community-based or clinical health or health care or healthcare or primary health or health service$ or medical or public health).ti,ab. |
| **Frameworks** | **7** | (framework* adj5 improv*).ti,ab |
|  | **8** | (framework* adj5 impact).ti,ab. |
|  | **9** | (framework* adj5 implem*).ti,ab. |
|  | **10** | (framework* adj5 accept*).ti,ab. |
|  | **11** | (framework* adj5 effect*).ti,ab. |
|  | **12** | (framework* adj5 qualit*).ti,ab. |
|  | **13** | (framework* adj5 optim*).ti,ab. |
|  | **14** | (framework* adj5 scal*).ti,ab. |
|  | **15** | (framework* adj5 transla*).ti,ab. |
|  | **16** | Or/7-15 |
| **All** | **17** | 5 and 6 and 16 |
| **Limits** | **18** | Limit 17 to English year 2003-current |

**Table 3.** Search terms (second search, additional terms in bold)

| **Improvement terms (Optimisation, QI, and CQI)** | **1** | (“quality improvement*” or “improve* quality” or “quality management*” or “improve* patient care” or “process improvement”).ti,ab. |
| --- | --- | --- |
|  | **2** | “Continuous Quality Improvement”.ti,ab. |
|  | **3** | “Total quality management”.ti,ab. |
|  | **4** | (“optimisation” or “optimization” or “optimi*”).ti,ab. |
|  | **5** | Or/1-4 |
| **Context** | **6** | (community or community-based or clinical health or health care or healthcare or primary health or health service$ or medical or public health **or chronic disease or smok* or tobacco or nutrition or diet or obesity or alcohol or physical activity or disease**).ti,ab. |
| **Frameworks** | **7** | (framework* adj5 improv*).ti,ab |
|  | **8** | (framework* adj5 impact).ti,ab. |
|  | **9** | (framework* adj5 implem*).ti,ab. |
|  | **10** | (framework* adj5 accept*).ti,ab. |
|  | **11** | (framework* adj5 effect*).ti,ab. |
|  | **12** | (framework* adj5 qualit*).ti,ab. |
|  | **13** | (framework* adj5 optim*).ti,ab. |
|  | **14** | (framework* adj5 scal*).ti,ab. |
|  | **15** | (framework* adj5 transla*).ti,ab. |
|  | **16** | **(framework* adj5 develop*).ti,ab.** |
|  | **17** | **(framework* adj5 intervention).ti,ab.** |
|  | **18** | Or/7-17 |
| **All** | **19** | 5 and 6 and 18 |
| **Limits** | **20** | Limit 19 to English year 2003-current |

**References**

1. Levati S, Campbell P, Frost R, et al. Optimisation of complex health interventions prior to a randomised controlled trial: a scoping review of strategies used. *Pilot and Feasibility Studies.* 2016;2(1):17.

2. Kaplan HC, Brady PW, Dritz MC, et al. The Influence of Context on Quality Improvement Success in Health Care: A Systematic Review of the Literature. *The Milbank Quarterly.* 2010;88(4):500-559.

3. Gardner K, Sibthorpe B, Chan M, Sargent G, Dowden M, McAullay D. Implementation of continuous quality improvement in Aboriginal and Torres Strait Islander primary health care in Australia: a scoping systematic review. *BMC health services research.* 2018;18(1):541.

4. Moullin JC, Sabater-Hernandez D, Fernandez-Llimos F, Benrimoj SI. A systematic review of implementation frameworks of innovations in healthcare and resulting generic implementation framework. *Health Res Policy Syst.* 2015;13:16.

5. Hempel S, Rubenstein LV, Shanman RM, et al. Identifying quality improvement intervention publications - A comparison of electronic search strategies. *Implementation Science.* 2011;6(1):85.
