# Supplementary figures and images for: Optimisation: defining and exploring a concept to enhance the impact of public health initiatives
Source: Health Res Policy Syst. 2019 Dec 30;17:108. doi: 10.1186/s12961-019-0502-6 (PMC6937822; doi:10.1186/s12961-019-0502-6)

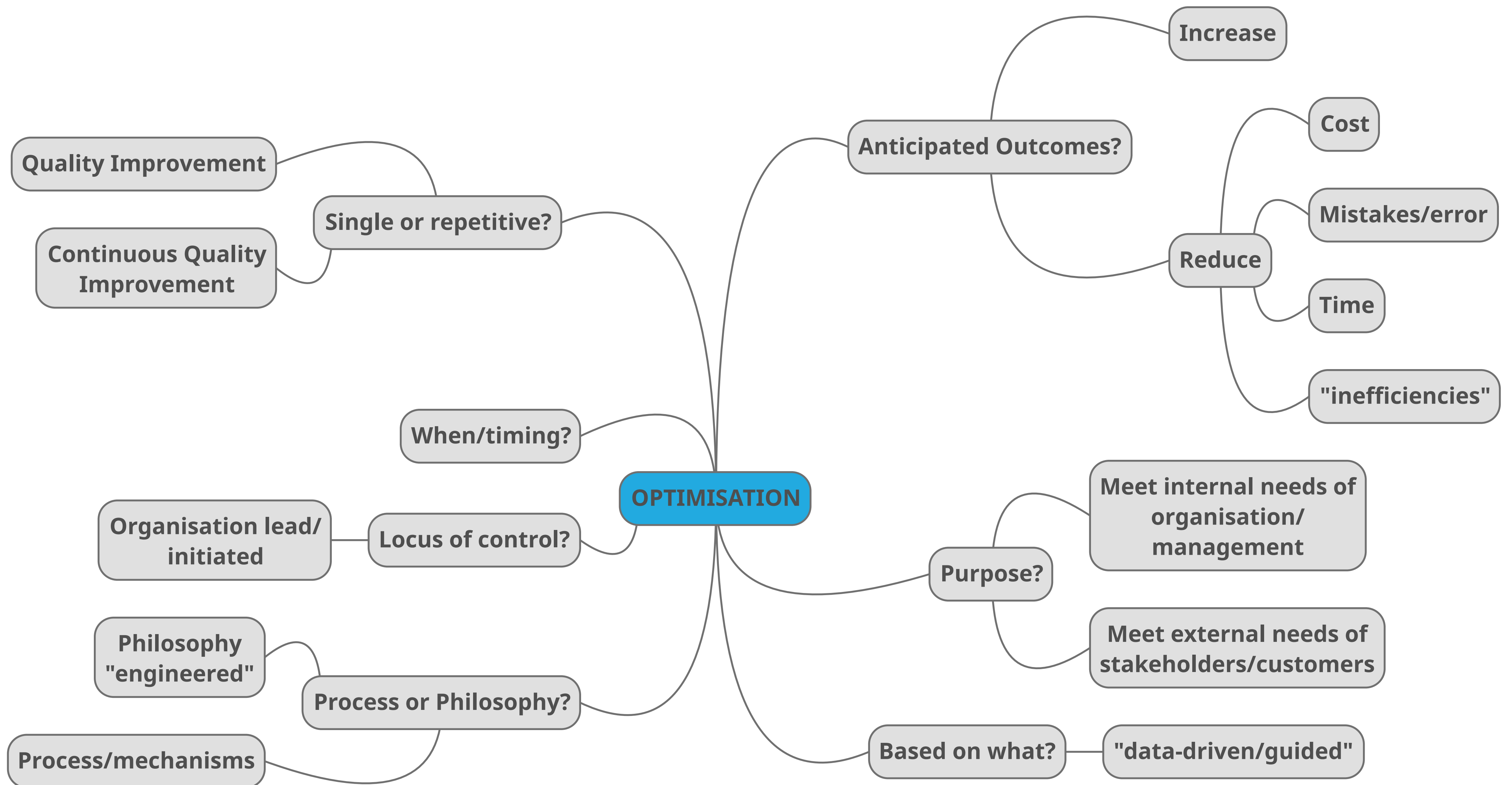

Supplement: Supplementary file 2 — Additional file 2. Conceptual map of 'Optimisation' derived from the literature. [file 12961_2019_502_MOESM2_ESM.pdf]

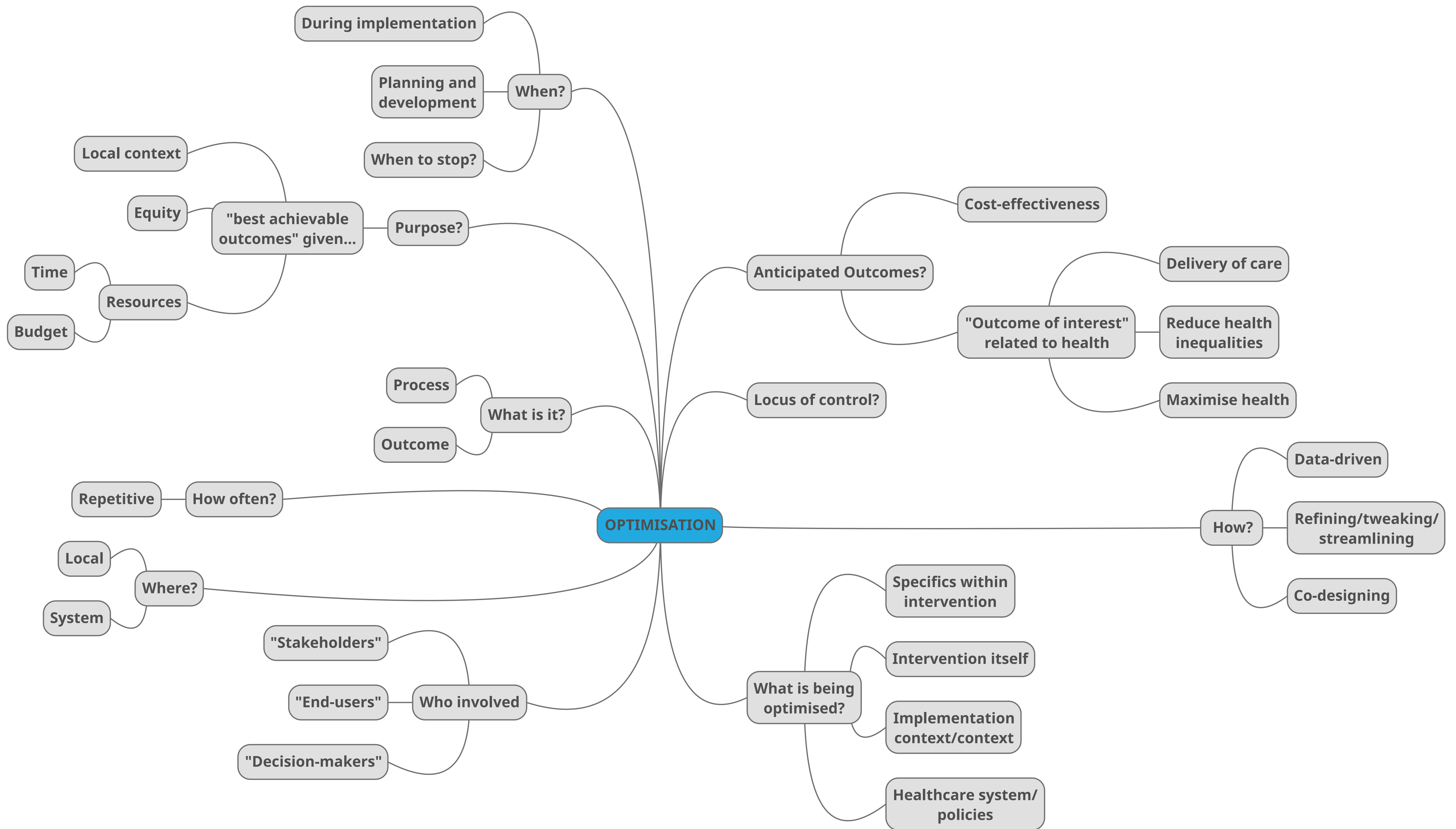

Supplement: Supplementary file 3 — Additional file 3. Conceptual map of 'Optimisation' derived from participant responses. [file 12961_2019_502_MOESM3_ESM.pdf]

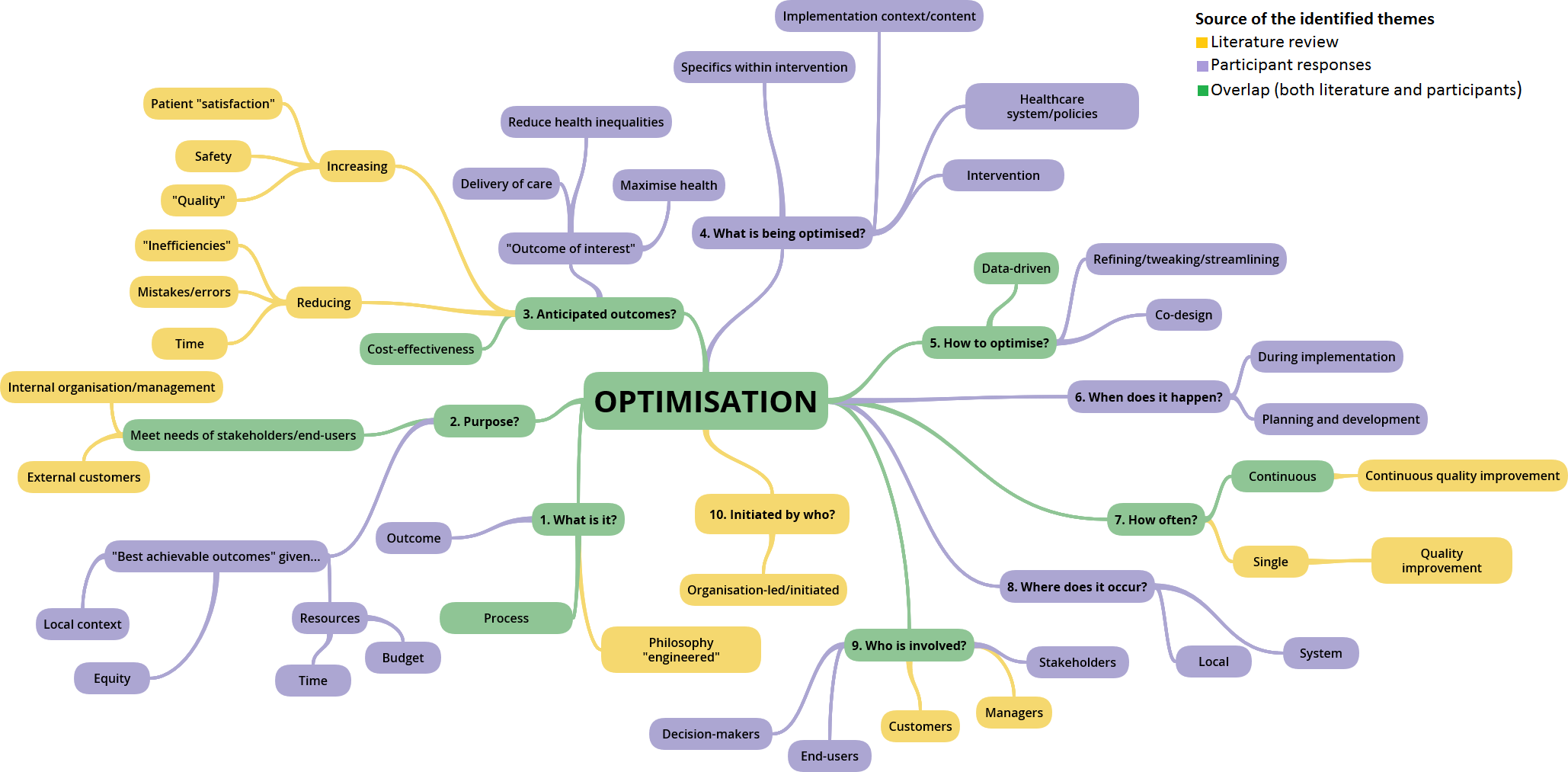

Supplement: Supplementary file 4 — Additional file 4. A combined conceptual map highlighting the overlap between two individual maps. [file 12961_2019_502_MOESM4_ESM.png]
